# Binding of NAD^+^-Glycohydrolase to Streptolysin O Stabilizes Both Toxins and Promotes Virulence of Group A *Streptococcus*

**DOI:** 10.1128/mBio.01382-17

**Published:** 2017-09-12

**Authors:** Jorge J. Velarde, Maghnus O’Seaghdha, Buket Baddal, Benedicte Bastiat-Sempe, Michael R. Wessels

**Affiliations:** Division of Infectious Diseases, Boston Children’s Hospital, and Department of Pediatrics, Harvard Medical School, Boston, Massachusetts, USA; University of Minnesota Medical School

**Keywords:** NAD^+^-glycohydrolase, *Streptococcus pyogenes*, cholesterol-dependent cytolysin, pore-forming toxins, streptolysin O

## Abstract

The globally dominant, invasive M1T1 strain of group A *Streptococcus* (GAS) harbors polymorphisms in the promoter region of an operon that contains the genes encoding streptolysin O (SLO) and NAD^+^-glycohydrolase (NADase), resulting in high-level expression of these toxins. While both toxins have been shown experimentally to contribute to pathogenesis, many GAS isolates lack detectable NADase activity. DNA sequencing of such strains has revealed that reduced or absent enzymatic activity can be associated with a variety of point mutations in *nga*, the gene encoding NADase; a commonly observed polymorphism associated with near-complete abrogation of activity is a substitution of aspartic acid for glycine at position 330 (G330D). However, *nga* has not been observed to contain early termination codons or mutations that would result in a truncated protein, even when the gene product contains missense mutations that abrogate enzymatic activity. It has been suggested that NADase that lacks NAD-glycohydrolase activity retains an as-yet-unidentified inherent cytotoxicity to mammalian cells and thus is still a potent virulence factor. We now show that expression of NADase, either enzymatically active or inactive, augments SLO-mediated toxicity for keratinocytes. In culture supernatants, SLO and NADase are mutually interdependent for protein stability. We demonstrate that the two proteins interact in solution and that both the translocation domain and catalytic domain of NADase are required for maximal binding between the two toxins. We conclude that binding of NADase to SLO stabilizes both toxins, thereby enhancing GAS virulence.

## IMPORTANCE

The global increase in invasive GAS infections in the 1980s was associated with the emergence of an M1T1 clone that harbors a 36-kb pathogenicity island, which codes for increased expression of toxins SLO and NADase. Polymorphisms in NADase that render it catalytically inactive can be detected in clinical isolates, including invasive strains. However, such isolates continue to produce full-length NADase. The rationale for this observation is not completely understood. This study characterizes the binding interaction between NADase and SLO and reports that the expression of each toxin is crucial for maximal expression and stability of the other. By this mechanism, the presence of both toxins increases toxicity to keratinocytes and is predicted to enhance GAS survival in the human host. These observations provide an explanation for conservation of full-length NADase expression even when it lacks enzymatic activity and suggest a critical role for binding of NADase to SLO in GAS pathogenesis.

## INTRODUCTION

*Streptococcus pyogenes* (group A *Streptococcus* [GAS]), the etiologic agent of streptococcal pharyngitis and local skin infections such as impetigo, as well as invasive infections such as necrotizing fasciitis and streptococcal toxic shock, remains an important public health threat (1, 2). Although our understanding of invasive infections is incomplete, crucial virulence factors that contribute to pathogenesis have been identified. Two GAS toxins shown to play a role in virulence are streptolysin O (SLO), a member of the cytolysin family of toxins, and NAD^+^-glycohydrolase (NADase) (3–10). In a study characterizing the emergence of the currently predominant invasive M1T1 strain, Nasser et al. (11) sequenced more than 3,600 isolates collected over approximately 40 years and found that, in the 1980s, a recombination event involving an M12 strain led to the incorporation of a 36-kb region of DNA, which included the genes for SLO (*slo*) and NADase (*nga*), into the M1 background. Among other changes, the recombination event introduced promoter polymorphisms that increased expression of both toxins (12, 13). This event was temporally associated with the emergence of the contemporary M1T1 lineage as the dominant clonal group among invasive GAS isolates. Both SLO and NADase, either alone or in combination, have been experimentally demonstrated to mediate host cell toxicity (3, 6, 7, 14–16).

SLO, a cholesterol-dependent cytolysin (CDC), acts, at least in part, by disruption of eukaryotic cell membranes. Like other CDCs, it oligomerizes on the surface of cells and inserts into the membrane to create a large pore (15). Sufficient damage to the cell membrane results in cell death, as demonstrated in studies using macrophages, neutrophils, keratinocytes, and red blood cells (6, 17–21). At sublytic concentrations, SLO has been shown to inhibit degranulation of neutrophils (19). SLO thus contributes to bacterial evasion of phagocytosis and resistance to killing by the immune system.

NADase cleaves NAD to produce nicotinamide and adenosine diphosphoribose in mammalian cells, thereby promoting cytotoxicity through depletion of energy stores (7, 8, 22). Its subunit architecture includes an N-terminal translocation domain and a C-terminal catalytic domain that harbors NADase activity (23). Deletions in the N-terminal translocation domain or C-terminal catalytic domain hinder SLO-mediated translocation of NADase into epithelial cells (24). On the other hand, deletions in the C terminus, but not the N terminus, inhibit enzymatic activity. The role of the translocation domain is not well understood, although data suggest that it interacts with the host cell surface (25).

SLO and NADase are known to be intimately associated with one another during infection. Madden et al. (9) first described the dependence of NADase on SLO for internalization into mammalian cells, a process that they termed cytolysin-mediated translocation (CMT). The details of NADase internalization are not completely understood, but it is thought that SLO mediates translocation of NADase across the cell membrane in a mechanism that is independent of pore formation (26). Several studies have also demonstrated synergistic toxicity by GAS infection when both toxins are present; derivatives with a deletion in either toxin, in both *in vitro* and *in vivo* models, are less cytotoxic (4–7, 16, 22, 27–29). Finally, the pathogen is better able to survive after internalization into keratinocytes in the presence of both SLO and NADase through inhibition of maturation of autophagosomes that contain intracellular GAS (6). Thus, a complete understanding of GAS pathogenesis requires consideration of the synergistic effects of SLO and NADase. Although the relationship between these two toxins appears to be critical for pathogenesis, the biochemical determinants of their interaction have not been well described.

The genes encoding NADase and SLO are located in an operon along with *ifs*, which encodes an intracellular NADase inhibitor (30), and expression of all three genes is regulated by a promoter upstream of *nga* (31). Both toxins are secreted, and they are thought to quickly interact with the mammalian cell surface and with each other. Indeed, Madden et al. (9) demonstrated that translocation of NADase by SLO is dependent on secretion of both proteins from the same bacterium and requires bacterial attachment to host cells. Interestingly, natural mutations that render NADase catalytically inactive can be found in clinical isolates of GAS. Among these, substitution of aspartate for glycine at position 330 (G330D) is a common inactivating polymorphism (10, 27, 32). Under these conditions, *ifs* becomes a pseudogene and is no longer properly expressed, suggesting that its function as an NADase inhibitor is dispensable in the absence of enzymatically active NADase (10, 30). However, the full-length NADase-inactive enzyme continues to be expressed. It is unclear why continued expression of inactive NADase undergoes positive selection. One possibility is that the enzymatically inactive protein retains some toxicity that is not yet fully understood (22, 27). Infecting A549 epithelial cells with a GAS M6 strain harboring catalytically inactive NADase was associated with increased toxicity compared to infection with strains harboring an NADase deletion (27). Inactive NADase has also been demonstrated to enhance Jun-N-terminal-protein-kinase (JNK)-mediated cell toxicity, a process that is dependent on SLO (22). However, Sharma et al. (14) directly introduced purified recombinant NADase into oropharyngeal keratinocytes independently of SLO using an anthrax toxin delivery system and demonstrated keratinocyte toxicity using the active toxin but no toxicity when the inactive G330D variant was used. One possible explanation for these findings is that the enhanced toxicity to host cells associated with the production of inactive NADase is not through a direct effect of this protein on the mammalian cell.

We reasoned that the positive selection of inactive NADase and the increased cytotoxicity of GAS that produces it could be a result of its effect on another virulence factor. Specifically, we studied the interaction of NADase with its cotoxin, SLO. We found that, after secretion of the two toxins from the bacterium, the abundances of SLO and full-length NADase were interdependent. We demonstrate a direct interaction of the two purified proteins in solution and show that both the N- and C-terminal domains of NADase contribute to its binding to SLO. Stabilization of SLO by its interaction with NADase in turn results in increased SLO-mediated toxicity to host cells. On the basis of these observations, we propose that enhancement of the abundance of SLO and its effects on cytotoxicity and intracellular survival provides an explanation for the conserved expression of full-length NADase among clinical isolates despite the presence of polymorphisms that attenuate or inactivate NADase enzymatic activity.

## RESULTS

### Full-length NADase supports SLO-mediated toxicity to keratinocytes.

**FIG 1:**
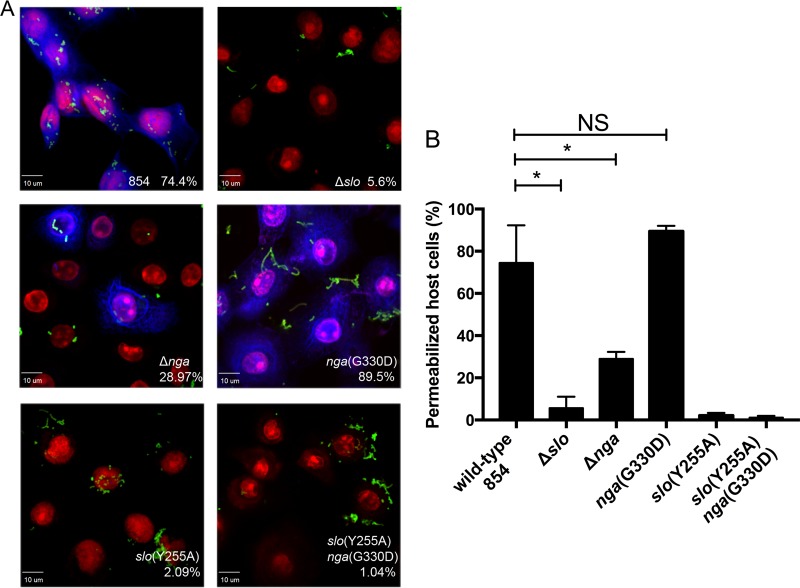
Inactive NADase can support SLO-mediated cell toxicity. (A) Cell membrane damage to human oropharyngeal keratinocytes due to SLO and NADase was assessed by uptake of a fixable viability dye (blue) after 1.5-h exposure to live GAS (green). Keratinocyte nuclei are stained with propidium iodide (red). The percentage of permeabilized cells is indicated for wild-type GAS 854 and isogenic mutant strains (Table 1 shows strain phenotypes). (B) Quantification of microscopic data is represented from three independent experiments. Data represent the mean ± standard deviation. The significance of differences between groups was evaluated by one-way analysis of variance with Tukey’s posttest (*, *P* < 0.05; NS, not significant).

Studies in human cell lines, including A549 and HeLa cells, have demonstrated that expression of the inactive variant NADase G330D from GAS confers additional toxicity to epithelial cells compared to an Δ*nga* derivative (22, 27). To further investigate the role of NADase in cytotoxicity, we compared the abilities of NADase-producing GAS and several mutants to cause membrane damage to primary human soft-palate keratinocytes (OKP7 cells) *in vitro*. After exposure of the cells to GAS, cytotoxicity was evaluated by microscopy to assess the cells’ ability to exclude a membrane-impermeant dye. Quantification of permeable and impermeable cells revealed that wild-type GAS, after a 1.5-h infection, caused 74.4% of cells to take up the dye. This effect was dependent on SLO, since only 5.6% of keratinocytes infected with the Δ*slo* derivative exhibited dye uptake. In contrast, 89.5% of cells treated with GAS producing the inactive NADase G330D variant became permeable, a significantly higher fraction than that observed for cells exposed to the GAS Δ*nga* strain (29%), which does not produce any NADase protein (Fig. 1).

It has been proposed that enzymatically inactive NADase protein retains some cytotoxic activity, which might explain the greater proportion of permeabilized cells observed after exposure to NADase G330D than after exposure to the Δ*nga* strain. To address this possibility, we tested the cytotoxic effects of GAS producing a variant of SLO that does not form pores but retains the ability to translocate NADase [*slo*(Y255A)] (26). Infection with the *slo*(Y255A) strain producing either wild-type NADase or enzymatically inactive NADase G330D caused minimal cytotoxicity (2.1% and 1.0% cell permeability, respectively) (Fig. 1). Since translocation of enzymatically active or inactive NADase failed to induce cytotoxicity in the absence of SLO pores, these data imply that the keratinocyte membrane permeabilization observed in our assay is driven by SLO. A further implication of these findings is that the increased level of SLO-dependent cytotoxicity observed in the presence of catalytically active or inactive NADase might reflect an effect of NADase on SLO.

### SLO is reduced in NADase-negative strains of GAS*.*

**FIG 2:**
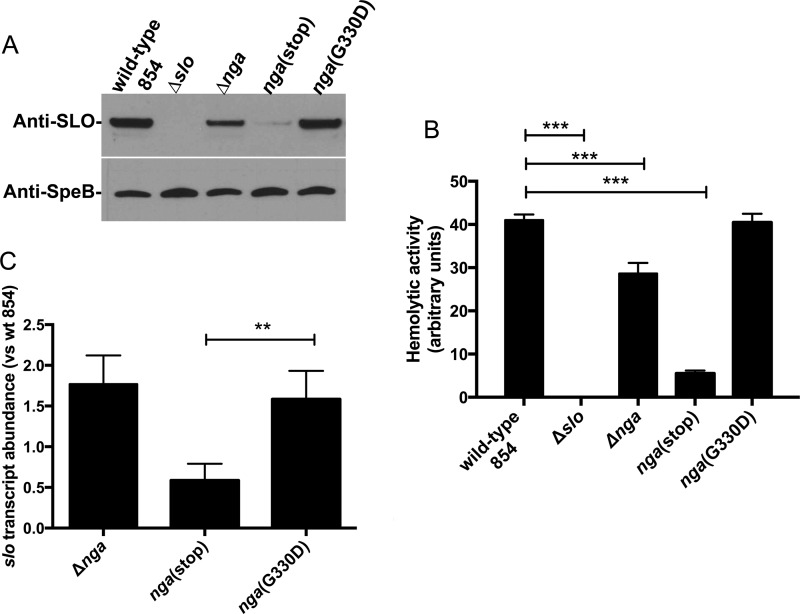
NADase supports SLO protein abundance. (A and B) The abundance of SLO was evaluated by Western blotting (A) and hemolytic activity (B) of culture supernatants of Δ*nga*, *nga*(stop), and *nga*(G330D) GAS strains; Table 1 shows strain phenotypes. (C) Transcript abundance of *slo* for GAS strain 854 derivatives. Data are normalized to the wild-type (wt) strain and represent the mean ± standard deviation from three experiments. The significance of differences between groups was evaluated by one-way analysis of variance with Tukey’s posttest (*, *P* < 0.05; **, *P* < 0.01; ***, *P* < 0.001).

We tested the hypothesis that deletion of NADase leads to a reduced amount of SLO in culture supernatants. The genes encoding NADase, its intracellular inhibitor Ifs, and SLO are arranged in a polycistronic operon in the order *nga*-*ifs*-*slo*, and the toxins are produced and secreted together. These proteins are most abundant in culture supernatant in late exponential to early stationary growth phase (33, 34), and prolonged incubation into late stationary growth phase is associated with degradation by the streptococcal cysteine protease SpeB (35). Wild-type GAS and Δ*slo* and Δ*nga* isogenic mutants were grown to late exponential growth phase, and culture supernatants were analyzed by Western blotting for the abundance of SLO using specific antiserum (Fig. 2A). Examination of the reactive bands by densitometry revealed that, relative to the wild-type parent strain, the amount of SLO was reduced by 44% in the Δ*nga* background. SLO hemolytic activity of culture supernatants (21) was also significantly reduced (by 30 to 40%) in supernatants from Δ*nga* mutants compared to the wild type (Fig. 2B).

Spontaneous mutations expected to prevent expression of full-length NADase include nonsense point mutations and small deletions or insertions that result in a frameshift and premature termination of translation. To test the effect of such a mutation on SLO abundance, we introduced a stop codon into the *nga* gene at codon 29 [*nga*(stop)]. This position is within the coding region of the NADase signal sequence, so no mature NADase protein is produced. Comparison of the abundance of SLO and SLO-hemolytic activity in the *nga*(stop) mutant revealed that both were significantly reduced with respect to the parental strain, even more so than in the Δ*nga* strain. Densitometric analysis of Western blots for SLO and measurement of hemolytic activity of culture supernatant indicated a reduction of 88.6% and 86.5%, respectively, in the *nga*(stop) mutant compared to the wild type (Fig. 2A and B). The amount of the protease SpeB, which is secreted in stationary phase but is not part of the *nga*-*ifs*-*slo* operon, was unchanged in the different GAS derivatives (Fig. 2A).

We next measured *slo* transcript in the Δ*nga* and *nga*(stop) strains to determine the contribution of polar effects of an NADase deletion on the transcript abundance of the downstream *slo* open reading frame. Quantitative reverse transcription-PCR (qRT-PCR) analysis revealed that the *slo* transcript was not reduced in the Δ*nga* strain compared to wild type. In the *nga*(stop) strain, there was a 41% decrease in *slo* transcript (Fig. 2C). Although this effect did not reach statistical significance by one-way analysis of variance comparison with wild-type 854, we did see a significant decrease compared to the *nga*(G330D) strain, suggesting a trend toward decreased *slo* transcript levels. We conclude from these data that nonsense mutations in *nga* can have downstream polar effects on *slo* transcript levels but that transcriptional effects may not fully account for the reduced level of SLO protein and hemolytic activity observed in *nga* mutants, since deletion of *nga* was also associated with a reduction in SLO protein but not with reduced *slo* transcript.

Because the cytotoxicity induced by GAS producing full-length but enzymatically inactive NADase was equivalent to that associated with wild-type GAS, we speculated that such a strain would produce wild-type amounts of SLO protein and hemolytic activity. To test this hypothesis, we characterized an isogenic mutant of wild-type strain 854 in which the chromosomal copy of *nga* codes for NADase harboring a G330D substitution. This strain, the *nga*(G330D) strain, produces similar levels of NADase as the wild type but has no detectable NADase enzymatic activity (3, 27). We found that the SLO protein abundance in the *nga*(G330D) strain was indistinguishable from that of the wild type and significantly greater than those of the Δ*nga* or *nga*(stop) strain (Fig. 2A and B). We conclude that the production of full-length NADase, either active or inactive, is associated with increased SLO abundance compared to that associated with the Δ*nga* or *nga*(stop) mutant.

### SLO protects NADase from proteolytic cleavage of its translocation domain.

**FIG 3:**
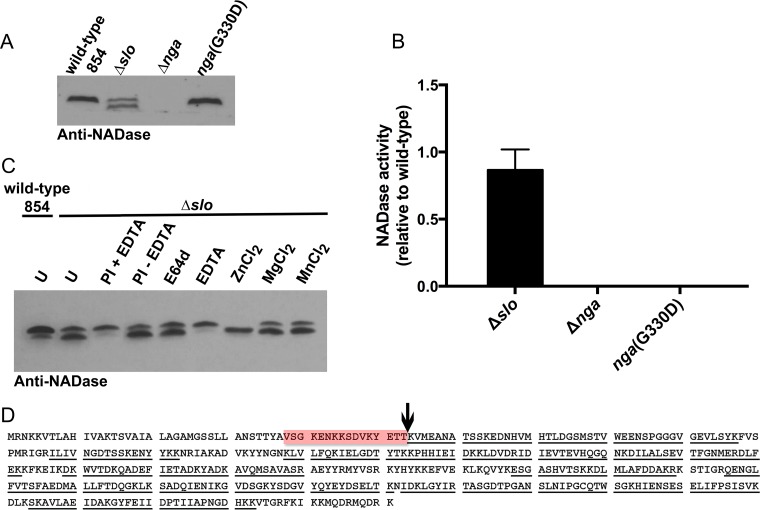
SLO prevents proteolytic cleavage of NADase. (A) Western blot using antiserum to NADase revealed two distinct bands in culture supernatant of a mutant strain deficient in SLO production (Δ*slo*) but not in supernatants from the wild type or a mutant producing NADase G330D. (B) NADase enzymatic activity associated with the Δ*slo* mutant was not significantly different from that of wild-type 854 as determined by one-way analysis of variance with Tukey’s posttest. (C) NADase cleavage in the Δ*slo* mutant is zinc dependent; it is inhibited by EDTA and enhanced by the addition of ZnCl_2_ (1 mM) but not MgCl_2_ (1 mM) or MnCl_2_ (1 mM). Experiments were performed three times, and where appropriate, the mean ± standard deviation is represented (U, untreated; PI, protease inhibitor). (D) The NADase cleavage site in the Δ*slo* mutant was determined by extraction of full-length and processed NADase from SDS-PAGE and analysis by tryptic digest and LC–MS-MS for fragment identification. The processing site was found to be between amino acids T53 and K54 (vertical arrow). The region of the protein identified in the full-length NADase but not the processed form is highlighted in red. Underlining denotes peptides identified in the analysis for both forms of NADase.

Since SLO stability is supported by the production of NADase (whether enzymatically active or inactive), we investigated whether NADase stability is reciprocally supported by the presence of SLO. Analysis of the culture supernatant of Δ*slo* cells revealed that deletion of SLO correlated with the appearance of a lower-molecular-weight species of NADase, although there was no effect on total NADase activity (Fig. 3A and B). Analysis of the full-length and truncated forms of NADase by mass spectrometry revealed a cleavage site between residues T53 and K54 in the N-terminal translocation domain (Fig. 3D). The fact that proteolysis occurs at a site remote from the C-terminal catalytic domain is consistent with the intact enzymatic activity of the processed species (24).

To investigate whether an extracellular protease is responsible for the differential processing of NADase in the Δ*slo* strain, wild-type or Δ*slo* GAS was grown in the presence of a protease inhibitor cocktail and NADase was analyzed by Western blotting. The presence of protease inhibitors correlated with the disappearance of the lower-molecular-weight species of NADase (Fig. 3C). Growth of the Δ*slo* strain in the presence of specific protease inhibitors revealed that NADase degradation was not due to the streptococcal cysteine protease SpeB, since it could not be inhibited using the cysteine protease inhibitor E64b (Fig. 3C). It is more likely that NADase is susceptible to cleavage by a zinc metalloprotease, since the divalent metal ion-chelating agent EDTA prevented NADase breakdown, and supplementation of the growth medium with Zn^2+^, but not Mg^2+^ or Mn^2+^, enhanced NADase degradation (Fig. 3C). Taken together, these data reveal an association between SLO and NADase that supports the stability and abundance of both proteins in the GAS extracellular milieu.

### SLO and NADase interact in solution as a heterodimer with 1:1 stoichiometry.

**FIG 4:**
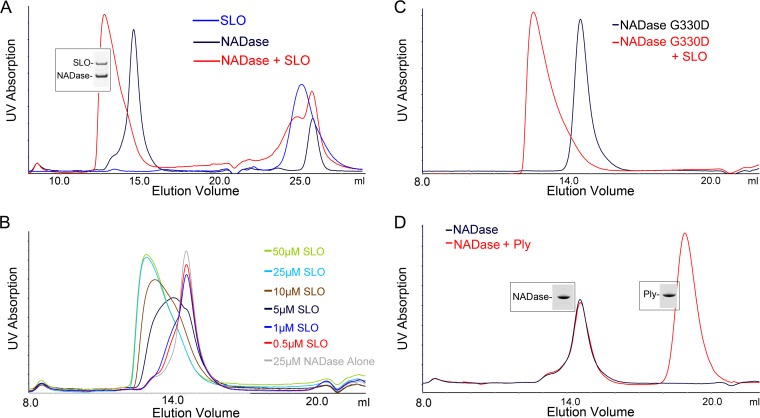
Analytical gel filtration reveals binding of NADase to SLO. (A) NADase and SLO were combined at final concentrations of 30 μM and were loaded on a Superdex 200 Increase 10/300 GL column. Both SLO and NADase could be recovered, as seen by Coomassie blue staining, from a peak that shifted to an earlier elution volume compared to that for either protein alone, suggesting an increase in size from NADase binding to SLO. (B) The shift in elution volume was concentration dependent, as demonstrated by keeping a constant concentration of NADase (25 μM) and incrementally increasing the SLO concentration. (C) A similar shift in elution volume was observed with SLO and the catalytically inactive NADase G330D. (D) Pneumolysin (Ply), a cytolysin from *S. pneumoniae*, failed to bind NADase at a 30 μM concentration, and thus, no shift for the NADase peak was observed. Representative chromatograms of at least three independent experiments are shown.

Our data suggested an interaction between SLO and NADase in the culture supernatant, likely after secretion of the toxins, leading to mutual protection from further processing or degradation. While a functional relationship between SLO and NADase is well established, the biochemistry of the binding interaction between these two toxins has not been well characterized. To address this, both toxins were purified and their interaction was investigated using analytical gel filtration. Purified NADase yielded a single symmetrical peak at an elution volume of 14.55 ml. Purified SLO eluted at a volume of 25 ml, which is larger than the column bed volume, suggesting that SLO interacts with the Superdex resin. However, when SLO and NADase were loaded on the column together at equal concentrations, the NADase peak shifted to an elution volume of 12.76 ml (Fig. 4A), and we could demonstrate coelution of both SLO and NADase, a result that suggests protein-protein interaction in solution. This effect was shown to be concentration dependent (Fig. 4B), and a similar interaction between SLO and the NADase G330D variant was observed (Fig. 4C). These data demonstrate an interaction between the two toxins in solution that does not require NADase to be enzymatically active. The column was calibrated with proteins of standard masses, and the Stokes radius of NADase alone and the NADase/SLO complex were determined to be consistent with globular protein masses of 59.5 kDa (predicted molecular mass, 47.2 kDa) and 136.3 kDa (predicted molecular mass of SLO + NADase, 110 kDa), respectively. To address the possibility that binding was nonspecific, we investigated whether NADase interacted similarly with another cholesterol-dependent cytolysin, pneumolysin from *Streptococcus pneumoniae*, which has 41.2% identity with SLO over domains 1 to 4. When NADase and pneumolysin were loaded on the column together, no shift in the NADase elution volume was observed, suggesting a lack of interaction with pneumolysin (Fig. 4D) and specificity of NADase binding to SLO.

**FIG 5:**
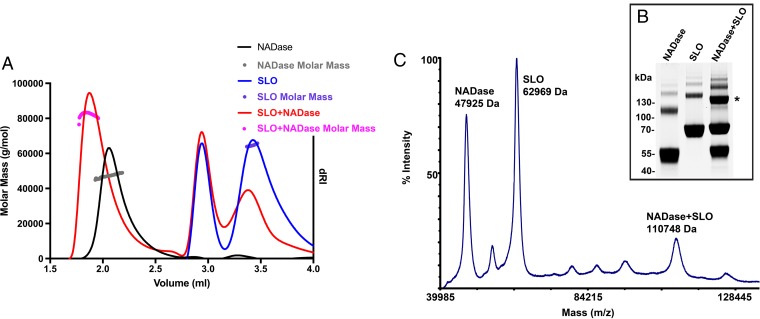
SLO and NADase bind in a 1:1 stoichiometry. (A) Molecular masses of NADase (47.2 kDa), SLO (64.6 kDa), and NADase + SLO (83 kDa) were determined by SEC-MALS of column elution peaks. Elution profiles are shown (solid lines) with calculated masses (circles) for each condition. (B and C) The stoichiometry of NADase-SLO binding was determined to be 1:1 by cross-linking of interacting NADase and SLO and identification of a heterodimer species by SDS-PAGE (*) (B) and MALDI-TOF mass spectrometry (C).

Because of the discrepancies between observed and expected elution volumes, particularly for SLO, we analyzed the molecular masses of each individual toxin and the combination of both by size exclusion chromatography coupled to multiangle light scattering (SEC-MALS), which is dependent on protein concentration and intensity of light scattering for accurate molecular mass determination irrespective of elution volume or protein shape (36). Analysis of elution peaks of each individual toxin (Fig. 5A) indicated an average molecular mass of 47.2 kDa for NADase (predicted value, 47.2 kDa) and 64.62 kDa for SLO (predicted value, 62.83 kDa). These results are consistent with expected molecular masses for monomer species of both NADase and SLO, in contrast to those estimated by elution volumes on Superdex 200. SEC-MALS analysis of the NADase/SLO complex at the same concentrations again revealed a shift in the NADase elution volume and a calculated average mass for the NADase/SLO complex of 83 kDa. This value is lower than the expected heterodimer mass (110 kDa), consistent with incomplete heterodimerization. Although these data are most consistent with a binding ratio of 1:1, they did not fully define the stoichiometry of binding between SLO and NADase. We therefore took a cross-linking approach to further investigate the stoichiometry of binding. After glutaraldehyde-cross-linking of an equimolar mixture of SLO and NADase, we identified, both by SDS-PAGE (Fig. 5B, asterisk) and by matrix-assisted laser desorption ionization–time of flight (MALDI-TOF) mass spectrometry (Fig. 5C), a major cross-linked species with a molecular mass of 110.7 kDa (Fig. 5C). This molecular species was absent in control cross-linking reaction mixtures containing either NADase or SLO alone (see Fig. S1 in the supplemental material), and it has a molecular size consistent with a heterodimer exhibiting 1:1 binding stoichiometry.

10.1128/mBio.01382-17.1 FIG S1  MALDI-TOF mass spectrometry of cross-linked NADase and SLO. Both NADase and SLO were individually cross-linked with glutaraldehyde for comparison to an experiment in which both proteins were present. NADase (A) and SLO (B) show molecular weights in agreement with predicted molecular weights. Some nonspecific dimerization can also be identified. Download FIG S1, PDF file, 0.3 MB. Copyright © 2017 Velarde et al. 2017 Velarde et al. This content is distributed under the terms of the Creative Commons Attribution 4.0 International license.

**FIG 6:**
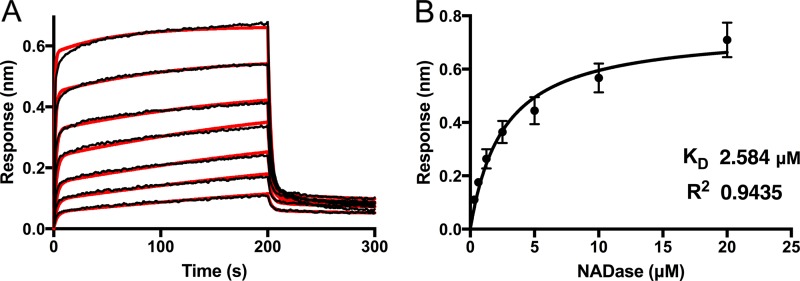
Biolayer interferometry (BLI) analysis of NADase binding to SLO. (A) Kinetic analyses of the interaction between full-length NADase and SLO were performed using biolayer interferometry. SLO was immobilized on a biosensor tip, and the binding of incremental 2-fold increases in concentrations of NADase from 312.5 nM to 20 μM was observed. The association and dissociation phases of the experiment are shown. Individual response curves are shown for increasing NADase concentrations. The data were fitted to a biphasic binding model (overlaid in red). (B) An overall dissociation constant (*K*_*D*_) of 2.58 μM ± 0.38 μM was determined from steady-state analysis of the BLI response versus NADase concentration determined late in the association phase. All experiments were repeated three times. Error bars represent standard deviations.

To further characterize binding between SLO and NADase, we used biolayer interferometry (BLI) to study the kinetics of interaction using a range of NADase concentrations from 312.5 nM to 20 μM (Fig. 6). The binding data could be fitted to a binding model with two phases. The second phase could represent nonspecific binding, or it could reflect two domains of NADase interacting with SLO or a conformational change induced by the initial interaction (Fig. 6A). Given this uncertainty, we chose to use steady-state analysis of binding at various concentrations of NADase to obtain a dissociation constant. We determined a *K*_*D*_ (equilibrium dissociation constant) of 2.6 ± 0.38 μM (Fig. 6B).

### The translocation domain of NADase is necessary, but not sufficient, for maximal SLO binding.

**FIG 7:**
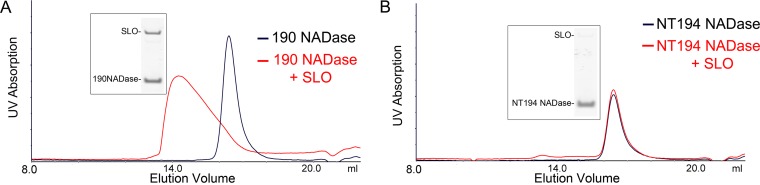
The C-terminal catalytic domain (190NADase) but not the N-terminal translocation domain (NT194NADase) binds independently to SLO as determined by analytical gel filtration. (A) Analytical gel filtration on a Superdex 200 Increase 10/300 GL column revealed a shift in the elution volume for the catalytic domain of NADase (190NADase) in the presence of SLO. SDS-PAGE and Coomassie blue staining of the peak fraction showed bands corresponding to both SLO and 190NADase. (B) A similar shift in elution volume was not seen for the translocation domain (NT194NADase) in the presence of SLO.

**FIG 8:**
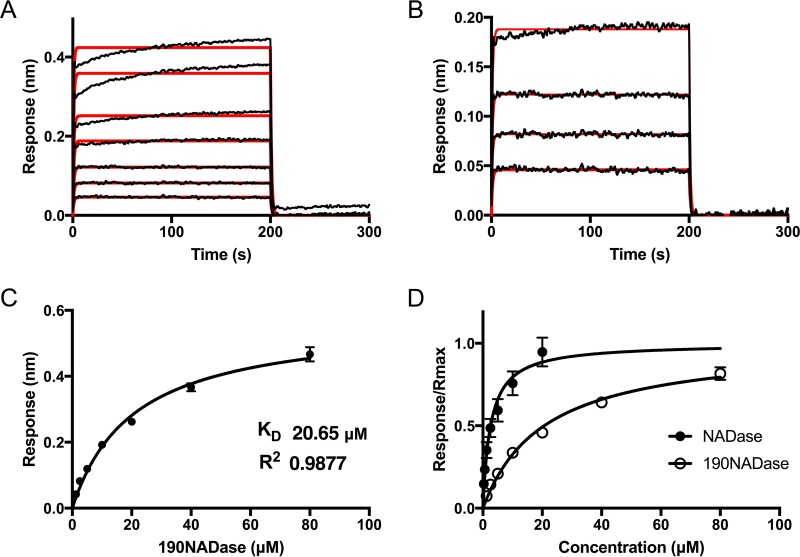
Kinetic analysis of 190NADase binding to SLO. SLO was immobilized on a biosensor tip, and the binding of 190NADase was assessed by BLI at incremental 2-fold increases in concentration. (A) Individual response curves are shown for 190NADase concentrations from 1.25 μM to 80 μM. The association and dissociation phases of the experiment are shown. The data fit a 1:1 binding model (red overlay) between 190NADase and SLO with a pattern suggestive of some nonspecific binding at the highest concentrations of 190NADase. (B) At concentrations of 1.25 to 10 μM 190NADase, a 1:1 binding model (red overlay) was in agreement with the data. (C) Steady-state analysis of the BLI response versus concentration of 190NADase yielded a *K*_*D*_ of 20.65 ± 1.70 μM. (D) A comparison of saturation curves for NADase and 190NADase demonstrates higher affinity between SLO and full-length NADase than between SLO and the 190NADase catalytic domain. All experiments were repeated three times. Error bars represent standard deviations.

NADase is composed of an N-terminal translocation domain and a C-terminal catalytic domain. To investigate which domain was responsible for binding to SLO, we expressed and purified the N-terminal translocation domain to residue 194 (NT194NADase) and, separately, the C-terminal glycohydrolase domain, beginning at residue 190 (190NADase). We repeated the analytical gel filtration experiments as described above, using the new constructs. Both 190NADase and NT194NADase alone exhibited an elution volume of 16.4 ml, a result consistent with a Stokes radius for a globular protein with a molecular mass of 25.4 kDa (predicted molecular masses, 30.15 kDa and 17.77 kDa, respectively). Our initial data (Fig. 3) suggested that the NADase translocation domain might contain the SLO binding site since this region is protected from further processing in the presence of SLO. Yet, no binding was observed between SLO and NT194NADase (Fig. 7B). However, we observed a shift in the elution volume of 190NADase in the presence of SLO (Fig. 7A) to an elution volume of 14.21 ml (molecular mass, 69.61 kDa; predicted molecular mass of SLO plus 190NADase, 92.89 kDa). Next, binding was investigated with BLI, using the same conditions as the experiments with full-length NADase (Fig. 8). The NT194NADase construct showed no interaction with SLO at concentrations up to 10 μM (data not shown). However, analysis of SLO binding by 190NADase and comparison to full-length toxin revealed a decreased affinity with a *K*_*D*_ of 20.6 ± 1.7 μM (Fig. 8C and D), according to steady-state analysis. The binding kinetics fit a 1:1 model at concentrations lower than the *K*_*D*_, although we noted some nonspecific binding at concentrations of ≥40 µM (Fig. 8A and B). Nevertheless, the 8-fold increase in *K*_*D*_ for the catalytic domain alone over full-length toxin, as determined by steady-state analysis, suggests participation of the N-terminal translocation domain of NADase in the binding interaction with full-length SLO (Fig. 8D). We conclude that both domains of NADase are necessary for the highest-affinity binding between the two toxins. These data are consistent with inhibition of further processing of NADase, at the translocation domain, presumably by interaction with SLO.

## DISCUSSION

Invasive GAS infections, for which SLO is a well-described virulence factor, continue to cause significant morbidity and mortality. Streptococcal toxic shock and necrotizing fasciitis have mortality rates as high as 44% and 32%, respectively, in the developed world, where medical care is readily available (37). Given the importance of SLO, we sought to better understand its biochemical relationship with NADase, a cotoxin that is expressed from the same operon (31). Previous work has demonstrated that NADase is dependent on SLO for translocation into eukaryotic cells, a phenomenon that has been termed cytolysin-mediated translocation (CMT) (9). It is also well described that many GAS strains, including some invasive isolates, harbor polymorphisms in the *nga* sequence that render NADase enzymatically inactive (10, 27). However, such strains invariably express full-length NADase protein despite these polymorphisms (10). Experiments investigating the potential toxicity of enzymatically inactive NADase G330D have suggested that there may be another, as-yet-uncharacterized, toxic effect of NADase that accounts for preserved expression of catalytically inactive NADase. Chandrasekaran and colleagues (22, 27) observed increased GAS toxicity to epithelial cells in the presence of enzymatically inactive NADase compared to strains harboring an NADase deletion and further suggested that inactive NADase could contribute to programmed cellular necrosis through the JNK pathway, a process that can also be mediated by SLO. In a separate study, Timmer et al. (17) demonstrated the induction of macrophage apoptosis due to intoxication by SLO. The authors reported a contributory role of NADase to this effect whereby GAS-induced toxicity was markedly reduced in Δ*nga* derivatives and restored upon complementation, but the specific contribution of NADase to the phenotype was not characterized. Furthermore, SLO-independent introduction of NADase G330D into keratinocytes using an anthrax toxin delivery system did not result in cell death, whereas delivery of wild-type toxin was lethal to keratinocytes (14). Together, these earlier observations led us to propose that NADase may enhance the cytotoxic effects of SLO even in the presence of polymorphisms that attenuate enzymatic activity. In this study, we tested that hypothesis by examining the physical and functional interactions between SLO and NADase.

We initially noted that the quantity of SLO in culture supernatants was dependent on expression of full-length NADase but was independent of NADase activity. Introduction of a premature termination codon in *nga* resulted in a significant reduction in SLO, an effect that could be attributed, in part, to a reduction in mRNA levels. Interestingly, as discussed above, nonsense or frameshift mutations resulting in premature termination are not seen in clinical isolates. Our results are consistent with strong positive selection for expression of full-length NADase, which appears to be required for optimal expression and stability of SLO. The polar effect from introduction of a stop codon in *nga* is consistent with a well-described Rho-dependent effect of a premature stop codon preventing transcription of downstream genes in a polycistronic operon (38). The gene encoding NusG, which has been demonstrated to participate in coupling of RNA polymerase and ribosomes in addition to Rho-dependent termination (38, 39), immediately precedes *nga* on the GAS chromosome (31), although the significance of its proximity to the *nga-ifs-slo* operon is not known. Thus, mutations that prevent expression of full-length NADase result in reduced abundance of SLO through both transcriptional and posttranscriptional mechanisms.

Conversely, expression of SLO appears to play a role in protecting NADase from proteolytic processing. Differential processing of the translocation domain of NADase in the absence of secreted SLO implies that physical interaction with SLO protects NADase from proteolysis. Although this effect has not been reported by other groups studying NADase in a *slo* deletion strain (16), multiple factors could contribute to detection of the proteolytic product, including, for instance, the extent of *slo* deletion, the epitope specificity of the antibody to NADase, or the growth phase of the bacterial cultures. We found that, in the absence of SLO, NADase undergoes proteolytic cleavage between residues 53 and 54 in the N-terminal translocation domain, an observation that implies that SLO is required not only to mediate translocation of NADase but also to protect a domain of NADase that is necessary for translocation to occur. Ghosh and Caparon (24) showed that even small deletions in the translocation domain resulted in defective cytolysin-mediated translocation. Interestingly, we found that differential processing of NADase at the translocation domain, in the absence of SLO, could be inhibited by EDTA and driven by zinc, suggesting that NADase cleavage could be mediated by a zinc metalloprotease that is extracellular to GAS. Susceptibility of the translocation domain to proteolysis in the absence of SLO may explain why both toxins must be secreted from the same bacterial cell, allowing for rapid association and subsequently for NADase translocation (9).

Toxicity of active NADase to epithelial cells and macrophages is well described (3, 7, 9, 14, 27). Furthermore, the role of NADase in prolonged survival of GAS that has been internalized in keratinocytes has been demonstrated (6). The differential processing that we observe for NADase would therefore have significant implications for GAS pathogenesis. Likewise, SLO has been identified as a major virulence factor (16, 29, 40), particularly for tissue injury and evasion of the innate immune system. SLO induces apoptosis in macrophages and neutrophils and at sublethal concentrations can inhibit degranulation of neutrophils during the innate immune response (17, 19). It is also directly toxic to keratinocytes (5, 29), as is once again demonstrated in our study. Reduction of SLO concentration, in the absence of NADase, would reduce SLO-mediated resistance to GAS killing by phagocytes and epithelial cells. Thus, the importance of SLO in persistence of GAS in the human host would result in positive selection of full-length NADase for its capacity to increase SLO abundance.

Our initial data regarding the mutual protection of SLO and NADase suggested that they must interact in solution after they are secreted from the bacterium. In an earlier study from our group, SLO bound to a solid support was shown to bind NADase in GAS culture supernatants, results that suggested direct interaction between the two toxins (29). In the present investigation, we used analytical gel filtration to detect binding in solution between SLO and full-length NADase, as well as binding of SLO to the catalytic domain of NADase (190NADase) at micromolar concentrations. Similar binding was not observed between NADase and pneumolysin. The latter result is consistent with earlier work in which another CDC, perfringolysin O, could not functionally replace SLO for NADase translocation (41). We used BLI to investigate in more detail the interaction between NADase and SLO. The affinity of binding between SLO and full-length NADase, as determined by BLI steady-state analysis, was 8-fold higher (*K*_*D*_, 2.584 μM) than the affinity of SLO for NADase lacking the N-terminal translocation domain (190NADase; *K*_*D*_, 20.65 μM). At micromolar concentrations, the N-terminal domain by itself did not bind to SLO. However, based on the difference in affinities between the C-terminal construct and full-length NADase, we conclude that both domains participate in binding between the toxins. BLI data for the interaction of full-length NADase with SLO fit a binding model with two phases. Such a binding pattern could represent nonspecific binding for the second phase. Another possible explanation is sequential binding of the translocation domain after initial interaction of the catalytic domain (190NADase), for which the data could be fit to a 1:1 binding model. Nevertheless, both the translocation domain and the catalytic domain must contribute to the overall binding affinity, as determined by steady-state analysis of the BLI data. These observations are consistent with previous observations by Ghosh and Caparon (24) suggesting that the entire NADase molecule is necessary for cytolysin-mediated translocation. We therefore propose that the interaction between SLO and NADase in solution stabilizes both toxins and represents an important early step of CMT.

The present investigation reveals that the concentration and stability of both SLO and NADase after secretion from the bacterium are each dependent on expression of the other toxin and that this reciprocal functional relationship is mediated, in part, by a direct binding interaction in solution between the two toxins. These results provide an explanation for the positive selection by GAS for expression of full-length NADase, even if the product lacks enzymatic activity. Our data suggest that the presence of either catalytically active or inactive full-length NADase leads to increased availability of SLO during pathogenesis and results in increased host cell toxicity. The ability of NADase to protect and maintain appropriate levels of secreted SLO during infection may be crucial to the pathogenesis of GAS and represents a critical relationship between two important virulence factors.

## MATERIALS AND METHODS

### Bacterial strains and growth conditions.

GAS 854 is an M-type 1 strain, isolated from a retroperitoneal abscess (42). The GAS strains used in this study are listed in Table 1. GAS was grown at 37°C in L3 or THY medium (Todd-Hewitt broth supplemented with yeast extract) as previously described or on Trypticase soy agar supplemented with 5% defibrinated sheep blood (Remel). *Escherichia coli* strain NEB5α (New England Biolabs) was used for cloning, and *E. coli* strain BL21 (Sigma) was used for protein expression unless otherwise noted. When necessary, antibiotics were added at the following concentrations: erythromycin was used at 1 µg/ml and 150 µg/ml for GAS and *E. coli*, respectively, and spectinomycin was used at 50 µg/ml in GAS or *E. coli*. For infection assays, penicillin was used at 20 µg/ml and gentamicin was used at 200 µg/ml. Ampicillin or carbenicillin was used at 100 µg/ml for *E. coli*.

**TABLE 1:** GAS strains used in this study

Strain	Genotype/properties	Reference
854	Wild-type M1 invasive isolate	42
854 Δ*slo*	*slo* deletion strain	3
854 Δ*nga*	*nga* deletion strain	3
854 *nga*(stop)	Stop codon at amino acid 29 of NADase	This study
854 *nga*(G330D)	Asp substitution at Gly330 of NADase, catalytically inactive	3
854 *slo*(Y255A)	Ala substitution at Tyr255 of SLO, inhibits pore formation but allows for NADase translocation	3
854 *slo*(Y255A)/*nga*(G330D)	Double mutant for inactive NADase and pore-deficient SLO	3

### DNA manipulation.

The *slo* gene was amplified by standard PCR of genomic GAS854 DNA using primers SLO_F (CTAGCTAGCGAATCGAACAAACAAAACACTGCT) and SLO_R (CGGCTCGAGCTACTTATAAGTAATCGAACCATA). Underlining in primer sequences indicates restriction endonuclease sites. The resulting product was digested with XhoI and NheI and ligated into vector pET28a linearized with the same enzymes. This construct contains the coding region for *slo* without a signal sequence and with an N-terminal 6×His tag; it was designated pETslo. For expression of the N-terminal translocation domain of NADase, a segment of *nga* corresponding to residues 38 to 194 was amplified by PCR, using pET*nga_ifs* (14) as a template, with primers NADaseNtermRB (AACCGGGATCCTTAATTGCCAAAGGTCACTTCGCTCAGAG) and NADaseNtermHRVFB (AACCGCCATGGGCCACCATCACCATCACCACGGCTCCCTGGAAGTGCTGTTTCAGGGCCCGGTCTCGGGCAAAGAAAACAAGAAATCGG). The product was digested with NcoI and BamHI and cloned into pET16a linearized with the same enzymes. The resulting clone pET_NADaseNterm_His_HRV harbors an N-terminal 6×His followed by a human rhinovirus (HRV) 3C protease site and the N terminus of NADase as outlined above. For expression of pneumolysin, the *ply* gene previously cloned into pQE30 was generously provided by Richard Malley (Boston Children’s Hospital) (43).

For generation of pNADase, the promoter and coding region for NADase and Ifs were amplified by PCR from strain 854 genomic DNA, using the primers Fw_P*nga* (5′ CCGGAATTCGACGGTGCCTTTATGGGACAAGAAGG 3′) and Rv_*ifs* (5′ GAGGCATGCCTAAAATGTTTCTATTGTTCTTTC 3′). This PCR product was cloned into the plasmid pDL278, using the restriction enzymes EcoRI and SphI.

Fidelity of all clones was confirmed by DNA sequencing.

### Generation of 854 *nga*(stop) strain by allelic exchange.

A stop codon in the *nga* gene corresponding to the amino acid at position 29 in NADase was introduced into the chromosome of GAS strain 854 by allelic exchange as previously described (44). Briefly, the stop codon was introduced using primers BBS67 (GGATCCGGCAGTGAAGCGTGTTTAC), BBS68 (GCTAATTAATTGCTACCCATTGATCAAGC), BBS69 (GCTTGATCAATGGGTAGCAATTAATTAGC), and BBS70 (GTCGACCAATCTCAATGTGATGCGG) by overlapping PCR with Easy-A polymerase (Agilent) and cloned into the pGEM-T Easy vector system (Promega). The product was then digested with BamHI and SalI and ligated into pJRS233. Allelic exchange was completed as previously described (44) in GAS strain 854. The mutation was confirmed by amplification and sequencing of the *nga* region of interest.

### Human cell culture, infection, and confocal microscopy.

OKP7 immortalized primary human soft-palate keratinocytes were a gift of James Rheinwald (45). Cells were cultured in keratinocyte serum-free medium (KSFM; Life Technologies, Inc.) as previously described (6, 46). For toxicity assays, sterile coverslips in 24-well plates were seeded with OKP7 cells and allowed to grow to near-confluence. GAS strain 854 and Δ*slo*, *nga*(stop), *nga*(G330D), *slo*(Y255A), and *slo*(Y255A)/*nga*(G330D) derivatives were added to separate coverslips at a multiplicity of infection (MOI) of 10 in KSFM and allowed to infect cells for 1.5 h at 37°C, 5% CO_2_. The cells were then washed three times with phosphate-buffered saline (PBS) and stained for viability using a fixable, Alexa Fluor 660-conjugated viability dye for 15 min in the dark (eBioscience). Cells were then washed again, followed by methanol fixation at −20°C. Next, fixed cells were stained with propidium iodide and anti-GAS group A carbohydrate antibody (47) fluorescently conjugated using the Alexa Fluor 488 antibody labeling kit (Pierce). Cells were washed between steps with PBS as before. Finally, coverslips were mounted using Prolong Gold (Thermo Fisher) and allowed to set for 16 h at ambient temperature in the dark. Samples were visualized by confocal microscopy using a Zeiss Axiovert 200 M inverted microscope at the Harvard Digestive Diseases Center Core Facility. Image acquisition and analysis were performed using SlideBook 6 software. The percent nonviable keratinocytes was calculated from at least 100 cells per experiment, and experiments were performed on three independent occasions. Statistical significance of differences between groups was determined using one-way analysis of variance with Tukey’s posttest (GraphPad Prism 7; GraphPad Software, Inc.).

### Culture supernatant immunoblot analysis.

GAS strains as listed in Table 1 were grown in L3 medium to late exponential phase (*A*_600_ of ∼0.8). Bacteria were harvested by centrifugation in a microcentrifuge, and supernatants were collected and sterile filtered. Samples of supernatant were separated by SDS-PAGE and transferred to nitrocellulose membranes. For experiments involving protease inhibitors and cations, the bacterial growth medium was supplemented as follows: cOmplete protease inhibitor cocktail with or without EDTA (Roche) was used according to the manufacturer’s instructions; EDTA, ZnCl_2_, MgCl_2_, and MnCl_2_ were each used at 1 mM; and E64d (Sigma) was used at 2 µM. Where appropriate, the following antisera were used: rabbit anti-NADase (1:1,000) and rabbit anti-SLO (1:1,000), both described previously (29), or rabbit anti-SpeB (1:1,000; Toxin Technology, Inc.). Horseradish peroxidase (HRP)-conjugated donkey anti-rabbit secondary antibodies (Santa Cruz Biotechnology) were used at a 1:5,000 dilution, and blots were developed using SuperSignal West Pico reagent (Pierce) and exposed to X-ray film. Comparison of toxin concentrations in culture supernatants was performed by densitometry using ImageJ software (NIH).

### SLO and NADase activity assays.

SLO activity was assayed by determination of hemolytic titer as previously described (21). GAS culture supernatants (150 μl) from late exponential or early stationary growth phase were used as a source of SLO. NADase assays were performed as previously described (5) using 100 μl of culture supernatant.

### RNA isolation and qRT-PCR.

GAS cultures were grown to mid-exponential phase (*A*_600_ of ∼0.5) in L3 medium. Isolation of total RNA was performed as described elsewhere (48). A NanoDrop ND-1000 spectrophotometer was used to evaluate the concentration and purity of isolated RNA. The QuantiTect SYBR green RT-PCR kit (Qiagen) was used to perform qRT-PCR on an ABI Prism 7300 real-time PCR system (Applied Biosystems). The following primers were used: for *slo*, rt-0167-F (5′ TACAAAACGACGTTGGGACA 3′) and rt-0167-R (5′ GATCACTTTTCGCCACCATT 3′), and for *recA*, rt-1800-F (5′ TGATTCTGGTGCGGTTGATC 3′) and rt1800-R (5′ ATTTACGCATGGCCTGACTC 3′). Primer annealing efficiency to a 4-log range of RNA template concentrations was 100% ± 5% for both primer sets. The expression level of *slo* was normalized to *recA* and analyzed using the threshold cycle (ΔΔ*C*_*T*_) method as described previously (48). Experiments were performed from three independent RNA preparations in triplicate. Statistical significance of differences between groups was determined using one-way analysis of variance with Tukey’s posttest (GraphPad Prism 7; GraphPad Software, Inc.).

### Identification of the NADase processing site.

GAS 854 *nga*(stop) complemented with pNADase, for overexpression of NADase, was cultured to late exponential phase (*A*_600_ = 0.9) in 10 ml of medium supplemented with 200 nM ZnCl_2_. Cells were removed by centrifugation, and the supernatant was sterile filtered and concentrated 20-fold in a Vivaspin 20 spin concentrator with a 10-kDa-molecular-mass cutoff (GE Healthcare). A 20-µl aliquot was then fractionated by SDS-PAGE. Two NADase bands that were absent from supernatants of the GAS 854 *nga*(stop) derivative of strain 854 were identified by Coomassie blue staining. The two bands were separately excised from the SDS-PAGE gel. These were provided to the Taplin Biological Mass Spectrometry Facility at Harvard Medical School for tryptic digest and liquid chromatography-tandem mass spectrometry (LC–MS-MS) analysis on an Orbitrap mass spectrometer (Thermo Fisher) using standard protocols.

### Protein expression and purification.

For purification of SLO, pETslo was transformed into *E. coli* BL21 for expression. Selected transformants were grown to an *A*_600_ of ∼0.8 to 1.0, and protein expression from the pET vector was induced at 25°C using IPTG (isopropyl-β-d-thiogalactopyranoside; 0.8 mM) for 4 h. Cells were harvested by centrifugation, lysed by addition of lysozyme (1 mg/ml) and DNase I (0.05 mg/ml), and subjected to sonication (Sonic Dismembrator; Thermo Fisher Scientific) in 20 mM Tris, 300 mM NaCl, 20 mM imidazole, 10% glycerol, 5 mM tris(2-carboxyethyl)phosphine (TCEP), supplemented with EDTA-free protease inhibitors (GE Healthcare). Lysates were purified over nickel-nitrilotriacetic acid (Ni-NTA) agarose (4-ml resin bed). The resin was washed with 20 column volumes of the lysis buffer without protease inhibitors, and protein was eluted using 350 mM imidazole in the same buffer. Fractions containing SLO were dialyzed into 20 mM Tris, 4 mM NaCl, 1 mM dithiothreitol (DTT), pH 8.0, and then diluted to a final NaCl concentration of 1 mM in the same buffer without NaCl. The sample was loaded on a HiTrap 5-ml Q Sepharose column in an Äkta purifier (GE Healthcare); washed with 5 column volumes of 20 mM Tris, 1 mM NaCl, 1 mM DTT, pH 7.8; and then eluted with a NaCl gradient to 300 mM over 20 column volumes. Fractions containing SLO were further dialyzed into PBS at pH 7.4, 1 mM DTT, 10% glycerol, and then separated on a Superdex 200 Increase 10/300 GL gel filtration column (GE Healthcare) in PBS containing 1 mM DTT. Activity of recombinant SLO was confirmed by hemolysis assay as described above.

Pneumolysin was expressed and purified in *E. coli msbB* as previously described (43), using 0.8 mM IPTG at 15°C for 14 to 16 h. Cells were lysed as described above in 20 mM Tris, 150 mM NaCl, pH 8.0, supplemented with EDTA-free protease inhibitors and 0.5 mM DTT. Cell lysate was cleared and loaded onto 1 ml of Ni-NTA resin, washed with 50 column volumes of the same Tris-NaCl buffer containing 20 mM imidazole, and then eluted with buffer containing 500 mM imidazole. The fraction with the highest concentration of protein was loaded onto a Superdex 200 Increase 10/300 GL column and separated with the same buffer containing no imidazole and 1 mM DTT at 0.5 ml/min.

Full-length NADase and the C-terminal 190NADase were purified as previously described (14).

The N-terminal NT194NADase construct was expressed by transforming pET_NADaseNterm_His_HRV into BL21 cells and inducing expression with 0.8 mM IPTG at an *A*_600_ of ∼0.5 to 0.8 at 30°C for 5 h. Cells were harvested and lysed by sonication in the presence of lysozyme and DNase I as described above in 20 mM Tris, 500 mM NaCl, 20 mM imidazole, pH 8.0, supplemented with EDTA-free protease inhibitors. The supernatant was cleared by centrifugation and filtration and then passed over 4 ml of Ni-NTA. The resin was washed with 25 column volumes of the same buffer and then eluted with 350 mM imidazole again in the same Tris buffer. Fractions containing protein were then pooled, and HRV 3C protease purified in house was added at a mass ratio of 1:100. Cleavage was allowed to proceed overnight at 4°C during dialysis into 20 mM Tris, 300 mM NaCl, 20 mM imidazole, 0.8 mM DTT, pH 8.0. The protein was then passed over a new 4-ml resin bed of Ni-NTA agarose, and the flowthrough along with 10 ml of wash with the same composition as the dialysis buffer was collected and analyzed for purity by SDS-PAGE.

All protein was rapidly frozen in a dry ice-ethanol bath and stored at −80°C for later use.

Protein concentration was determined by measuring *A*_280_ on a NanoDrop ND-1000 spectrometer (Thermo Fisher Scientific).

### Analytical gel filtration and size exclusion chromatography-multiangle light scattering (SEC-MALS).

NADase, 190NADase, and NT194NADase, either alone or in combination with SLO or pneumolysin, were mixed in PBS, 1 mM DTT, pH 7.4, at 25 to 30 μM concentrations. Aliquots of 100 μl were injected into an Äkta purifier fast protein liquid chromatography (FPLC) system with a Superdex 200 Increase 10/300 GL column (GE Healthcare). Proteins were analyzed on the Superdex column at a rate of 0.5 ml/min at 4°C. The resulting elution volumes for the mixture of NADase variants with SLO or pneumolysin were compared to elution volumes for NADase alone. For analysis of concentration-dependent changes, NADase was held at 25 μM and SLO was increased from 0 to 50 μM and again analyzed as described above. The column was calibrated for molecular mass of globular proteins using standards that included blue dextran (void), ferritin (440 kDa), aldolase (158 kDa), conalbumin (75 kDa), ovalbumin (44 kDa), RNase A (13.7 kDa), and aprotinin (6.5 kDa). Molecular masses were determined from protein standards using published methods (49, 50). Chromatogram images were created using Unicorn 5.11 and Adobe Illustrator CS5.1.

SEC-MALS (36) was used to determine the molecular mass of protein species. Fifty-microliter aliquots of 25 μM SLO, full-length NADase, or SLO plus NADase (25 μM each) in PBS, 1 mM DTT, pH 7.4, were loaded onto a Superdex 200 Increase 10/150 GL column (GE Healthcare) using an Agilent 1260 Infinity isocratic liquid chromatography system with an autosampler and in-line degasser. High-pressure liquid chromatography (HPLC) was coupled with an Optilab T-rEX refractive index detector (Wyatt) and a Dawn Heleos II multiangle light-scattering detector (Wyatt). The column was equilibrated, and all analyses were completed in PBS, 1 mM DTT, pH 7.4. We used 50 μg bovine serum albumin (BSA) for normalization of detectors, peak alignment, and correction for band broadening prior to experimental analysis. Experiments were completed at a rate of 0.4 ml/min at room temperature. Molecular mass determination based on light scattering intensity and measured protein concentration was performed with ASTRA 7 (Wyatt). Molecular masses were determined at least twice for each condition with similar results.

### Heterodimer cross-linking and MALDI-TOF mass spectrometry.

NADase, SLO, or NADase plus SLO, each at concentrations of 12 μM in PBS, 1 mM DTT, pH 7.4, was cross-linked with 0.005% glutaraldehyde for 10 min at room temperature. Reactions were stopped with Laemmli buffer and analyzed by SDS-PAGE. For MALDI-TOF mass spectrometry analysis, the reaction volume was 500 μl; reactions were stopped after 10 min with addition of Tris to 100 mM and then concentrated and buffer exchanged into 20 mM Tris, pH 6.8, using a spin concentrator (molecular weight cutoff [MWCO], 10,000; Pierce). The samples were then diluted 1:10 in 0.1% trifluoroacetic acid (TFA) and spotted with α-cyano-4-hydroxycinnamic acid (CHCA) on an Opti-TOF 384-well insert (AB Sciex). Masses were determined using standard protocols (51) on an ABI 4800 MALDI-TOF/TOF analyzer (Applied Biosystems).

### BLI.

Purified NADase and 190NADase were prepared for BLI (52) by dialyzing at 4°C into 20 mM Tris, 300 mM NaCl, 1 mM DTT, 0.05% Tween 20, pH 7.4. Protein concentration was determined by measuring *A*_280_ on a NanoDrop ND-1000 spectrometer. The initial concentration for NADase was 20 μM with subsequent 2-fold dilutions in a 96-well plate to a final concentration of 312.5 nM. Based on initial estimates of *K*_*D*_ for 190NADase, the highest concentration used for the experiment was 80 μM with 2-fold dilutions to 1.25 μM. SLO was biotinylated using EZ Link sulfo-*N*-hydroxysuccinimide (NHS)–LC-LC–biotin (Thermo Scientific) per the manufacturer’s recommendations. The BLI experiments were performed on an Octet RED384 system using streptavidin-coated sensor tips for kinetic studies (FortéBio) at 30°C with a shake speed of 1,000 rpm. Biosensor loading was performed with an SLO concentration of 50 nM for 200 s. The tips were then quenched in 10 μg/ml biocytin for 120 s, and a new baseline was obtained in buffer over 60 s. Association occurred in buffer containing NADase or 190NADase with concentrations described above, for 200 s followed by a dissociation phase in buffer alone for 180 s. Data were double subtracted using background from SLO-negative experiments at each NADase or 190NADase concentration and with NADase-/190NADase-negative experiments. Interstep corrections were applied. The data were fitted by nonlinear regression using GraphPad or the Octet data analysis software version 9.0. *K*_*D*_ calculations were based on steady-state response curves. Responses in nanometers at saturation between seconds 195 and 200 of the association phase were plotted against concentrations of NADase or 190NADase resulting in hyperbolic binding curves. *K*_*D*_ values were determined as the NADase or 190NADase concentrations at which 50% of the maximal response was achieved, which represents 50% binding of SLO on the sensor tip. All steps and dilutions during BLI analysis were performed in 200 μl of 20 mM Tris, 300 mM NaCl, 0.01% Tween 20, 1 mM DTT, pH 7.4.

For analysis of NT194NADase, protein was initially diluted 20-fold from its purification buffer to a starting concentration of 10 μM. Optimization trials with this construct in the above buffer using BSA at 0.1% showed no response during the association phase. BLI analysis of this construct was not pursued further.
