# Supplementary material for: Binding of NAD+-Glycohydrolase to Streptolysin O Stabilizes Both Toxins and Promotes Virulence of Group A Streptococcus
Source: mBio. 2017 Sep 12;8(5):e01382-17. doi: 10.1128/mBio.01382-17 (PMC5596348; doi:10.1128/mBio.01382-17)
Supplement: FIG S1 [file mbo004173476sf1.pdf]

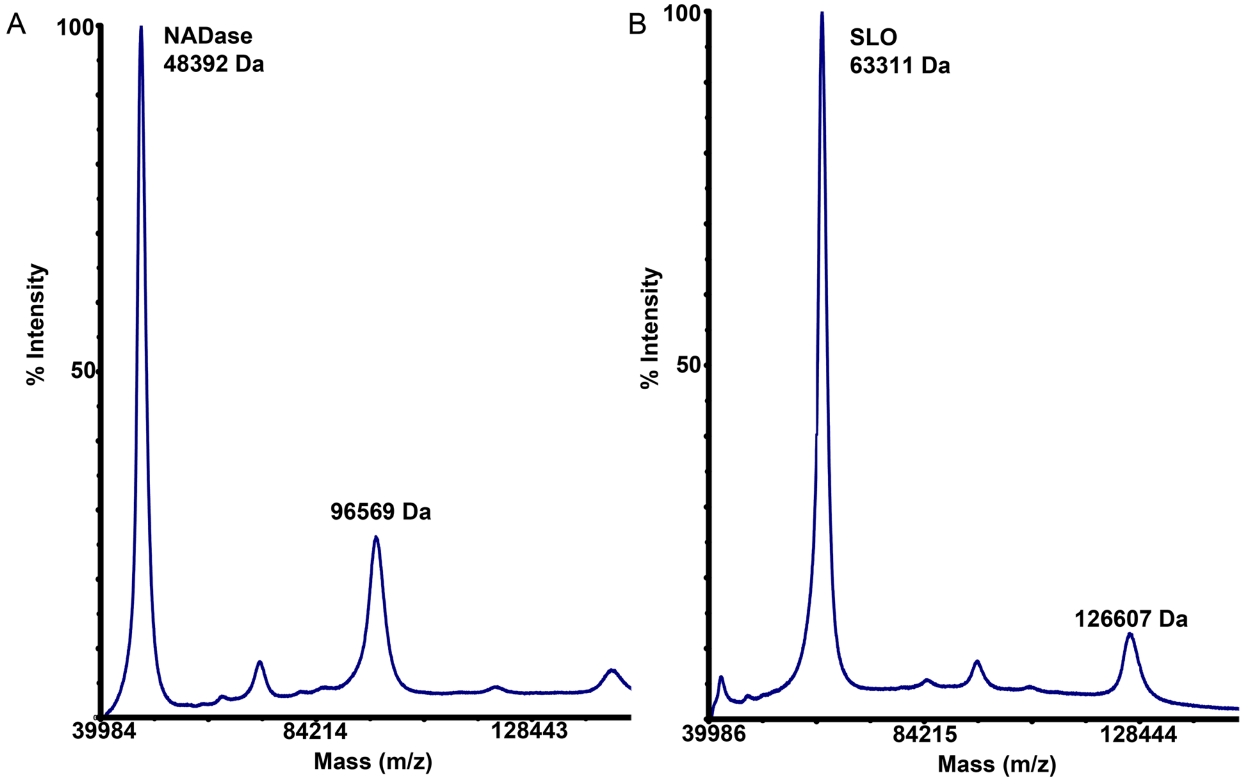


**Supplemental Figure 1. MALDI-TOF mass spectrometry of crosslinked NADase and SLO.** Both NADase and SLO were individually crosslinked with glutaraldehyde for comparison to an experiment in which both proteins were present. NADase (A) and SLO (B) show molecular weights in agreement with predicted molecular weights. Some nonspecific dimerization can also be identified.
